# Genetically detoxified tetanus toxin as a vaccine and conjugate carrier protein

**DOI:** 10.1016/j.vaccine.2022.07.011

**Published:** 2022-07-22

**Authors:** Min-Ju Chang, Morgane Ollivault-Shiflett, Richard Schuman, Son Ngoc Nguyen, Igor A. Kaltashov, Cedric Bobst, Shalini P. Rajagopal, Amanda Przedpelski, Joseph T. Barbieri, Andrew Lees

**Affiliations:** a Fina Biosolutions LLC, 9430 Key West Ave, Suite 200, Rockville, MD 20850, United States; b Antibody and Immunoassay Consultants, 9430 Key West Ave, Suite 201, Rockville, MD 20850, United States; c University of Massachusetts, 240 Thatcher Way, Life Science Laboratories N369, Amherst, MA 01003, United States; d National Institute for Biological Standards and Control, Medicines and Healthcare products Regulatory Agency, Blanche Lane, South Mimms, Potters Bar EN6 3QG, UK; e Medical College of Wisconsin, 8701 Watertown Plank Rd., Microbiology and Immunology BSB-2830, Milwaukee, WI 53226, United States

## Abstract

Tetanus toxoid (TTxd), developed over 100 years ago, is a clinically effective, legacy vaccine against tetanus. Due to the extreme potency of native tetanus toxin, manufacturing and regulatory efforts often focus on TTxd production, standardization, and safety, rather than product modernization. Recently, a genetically detoxified, full-length tetanus toxin protein (8MTT) was reported as a tetanus vaccine alternative to TTxd (Przedpelski et al. mBio, 2020). Here we describe the production of 8MTT in Gor/Met^™^ E. coli, a strain engineered to have an oxidative cytoplasm, allowing for the expression of soluble, disulfide-bonded proteins. The strain was also designed to efficiently cleave *N*-terminal methionine, the obligatory start amino acid for E. coli expressed proteins. 8MTT was purified as a soluble protein from the cytoplasm in a two-column protocol to > 99 % purity, yielding 0.5 g of purified 8MTT/liter of fermentation broth with low endotoxin contamination, and antigenic purity of 3500 Lf/mg protein nitrogen. Mouse immunizations showed 8MTT to be an immunogenic vaccine and effective as a carrier protein for peptide and polysaccharide conjugates. These studies validate 8MTT as commercially viable and, unlike the heterogenous tetanus toxoid, a uniform carrier protein for conjugate vaccines. The development of a recombinant, genetically detoxified toxin produced in *E. coli* aligns the tetanus vaccine with modern manufacturing, regulatory, standardization, and safety requirements.

## 1. Introduction

An effective vaccine against tetanus toxin (TT) has been available since the 1920 s and has reduced tetanus fatalities by 99 % [1,2]. While the general population in developed countries is vaccinated with chemically inactivated tetanus toxoid (TTxd), tetanus protection remains a critical issue and a global concern in developing countries [3-7]. According to WHO estimates, 34,000 and 25,000 neonates died from tetanus in 2015 [8] and in 2018 [9], respectively. In a position paper, WHO identified maternal and neonatal tetanus as a public health problem where immunization programs are suboptimal [6].

Although an inexpensive vaccine, TTxd manufacturing has several limitations, including the need for a dedicated biosafety level manufacturing facility, old production protocols, low purity, complex standardization, and local immune reactivity [10-13]. Commercial TTxd comprises 20–70 % toxoid with hundreds of *C. tetani* protein contaminants [10]. While another toxoided vaccine based on diphtheria toxin has been genetically detoxified (CRM_197_), there has been no equivalent for tetanus, which is a larger and more complicated toxin.

Carrier proteins are used to enhance the immunogenicity of peptides, glycans and other poorly immunogenic haptens. Only a few carrier proteins are approved for human use, which include TTxd and diphtheria toxoid (DTxd) and CRM_197_ [14,15]. Crude TTxd preparations need additional purification to achieve conjugation grade purity. TTxd is one of the few powerful carrier proteins in conjugate vaccines, with several pan T cell peptides (universal T cell epitopes) [16], which may improve TTxd effectiveness as a carrier. In contrast, CRM_197_ has fewer and weaker T cell epitopes than TTxd [16].

**Fig. 1.**
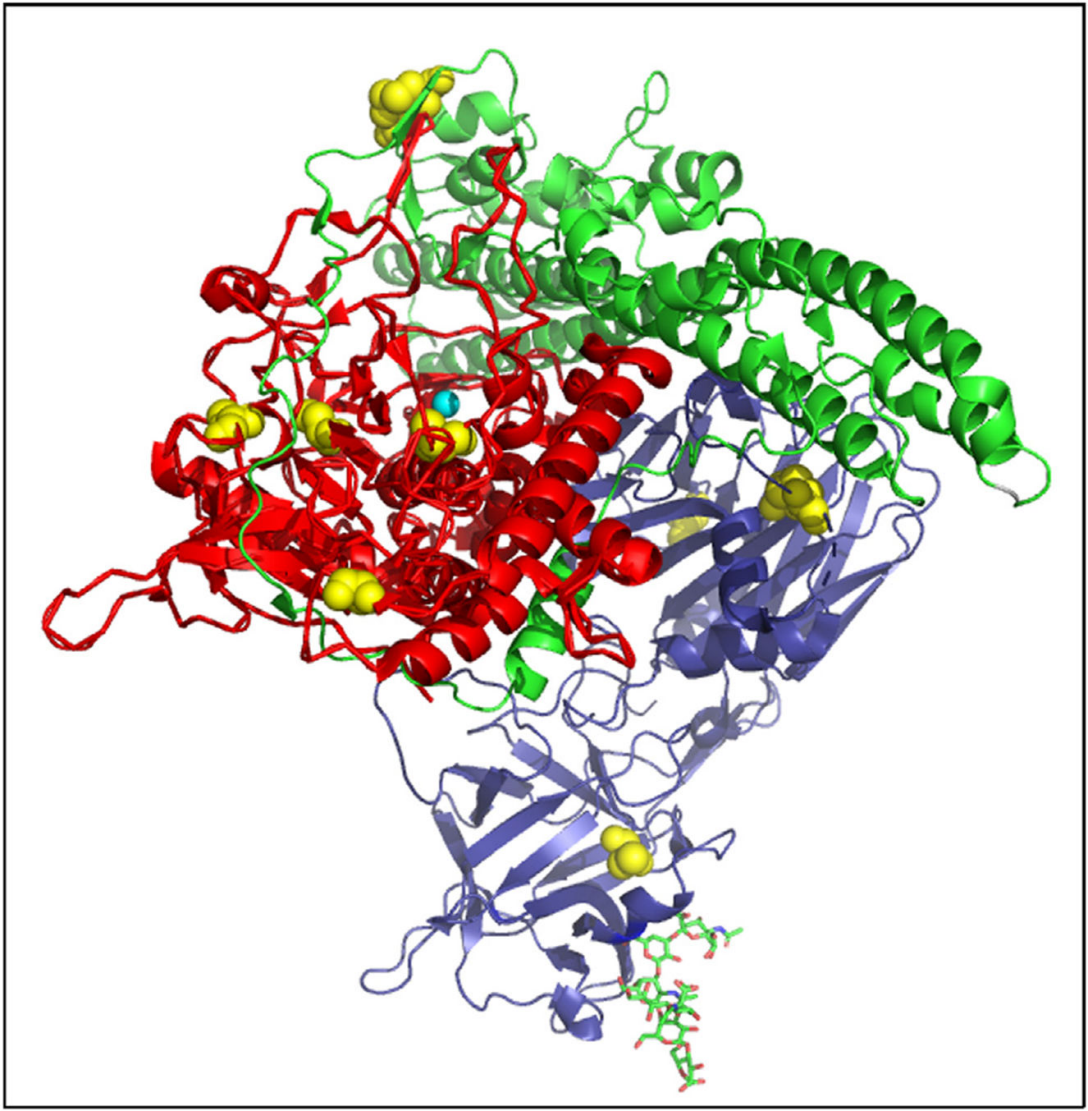
8MTT, an eight amino acid mutated form of Tetanus toxin The eight amino acids mutations in 8MTT were engineered to inactivate the three facets of tetanus toxin action: Light Chain catalysis (LC; red), substrate-binding Y26A, substrate cleavage L230K, zinc binding E234Q, R372A, and Y375F; Heavy Chain translocation (HCN; green), K768A; and Heavy Chain receptor binding (HCC; blue), R1226L and W1289A. Shown is the crystal structure of TT(RY), a 2MTT derivative of 8MTT, highlighting the ten cysteines (yellow), six free and four in disulfide bonds and Zinc (cyan) from PDB:5N0B.

Earlier studies showed the generation of full-length tetanus toxin with two-point mutations that reduced catalysis and toxicity by ~ 125,000-fold relative to native tetanus toxin [17,18]. More recently, a single amino acid mutation was shown to reduce Light Chain translocation [19], which allowed the development of a recombinant full-length tetanus toxin with eight individual amino acid mutations (8MTT) that inactivated catalysis, Light Chain translocation, and host receptor binding, while retaining 99 % amino acid identity with native tetanus toxin [20] (Fig. 1). Inactivation of multiple, independent biological functions provides a fail-safe basis for eliminating genetic reversion to toxicity, a property that is absolutely needed for a genetically engineered vaccine. 8MTT with a His-tag (His-8MTT) was expressed in the cytoplasm of *E. coli* grown in a shake flask. His-8MTT (single chain or trypsin nicked) was not toxic for outbred mice at a dose of 0.6 mg, making His-8MTT > 50 millionfold less toxic than native tetanus toxin [20]. His-8MTT elicited a robust immune response and good vaccine potency against native tetanus toxin challenge [20]. The current study describes the development of 8MTT as a vaccine and conjugate vaccine platform by fermentation expression in an engineered *E. coli* strain, development of an efficient 2-column purification scheme, and evaluation as a tetanus vaccine and conjugate vaccine carrier. This work facilitates the transition of tetanus vaccine from a chemical toxoid to a modern recombinant vaccine.

## 2. Results

### Optimization of 8MTT fermentation expression in Gor/Met^™^ *E. coli*

Tetanus toxin is a 1315 amino acid single- or di- chain protein with 10 cysteines, 2 sets of disulfide bonds and 6 unpaired thiols (Swiss-Prot: P04958.2) produced as a soluble monomeric protein when expressed in batch culture of *E. coli* BL21(DE3), Ni-affinity/size exclusion chromatography yielded ~ 5 mg of purified 8MTT-His / L of culture [20]. To enhance expression to levels suitable for commercialization, the recently developed Gor/Met^™^ *E. coli* strain [21] was used for 8MTT expression. Gor/Met^™^ *E. coli* expresses soluble, properly folded, disulfide-bonded proteins in the cytoplasm and cleaves the *N*-terminal methionine from newly synthesized proteins, Gor/Met^™^ E. coli was engineered by deleting the glutathione reductase gene (gor) to create an oxidative cytoplasm which allows for the expression of soluble, disulfide bonded proteins [22] and subsequently inserting the gene encoding methionine aminopeptidase into the gor-locus expressed under the control of a *tac* promoter [23]. The gene encoding 8MTT, without the His-tag, was subcloned into Gor/Met^™^ *E. coli* onto a plasmid under the control of a *tac* promoter (p8MTT). Thus, IPTG induces 8MTT expression along with the methionine aminopeptidase.

8MTT expression was initially evaluated in shake flasks with IPTG induction between 20 °C and 37 °C. Gor/Met^™^ *E. coli* (p8MTT) grew to a density of about 10 OD600 in Terrific Broth media, but with yields of < 20 mg of 8MTT /L of culture. We hypothesized that enhanced 8MTT expression in Gor/Met^™^ *E. coli* required more oxygen than could be provided in a shake flask. Therefore, further optimization of 8MTT expression was performed in a 5 L fermenter, using chemically defined media supplemented with yeast extract + glucose. High expression of soluble 8MTT was observed with IPTG induction between 22 °C and 28 °C, with Gor/Met^™^ *E. coli* (p8MTT) achieving OD600 densities of 10–100, corresponding to 13–100 g wet cell paste/L fermentation culture, depending on the inducing temperature. Cell productivity (mg 8MTT / g cell paste) was maintained regardless of cell density, *i.e.*, 8MTT yield was proportional to cell density. By SDS-PAGE, the highest cell density yielded ~ 1 g of 8MTT / L fermentation culture.

### Purification of 8MTT

Following harvest of the cell mass, subsequent homogenization, and clarification, 8MTT was captured from the lysate onto an anion exchange resin with dextran extender tentacles. 8MTT from the eluant of the anion exchange resin was brought to 2 M NaCl and loaded onto a butyl hydrophobic interaction chromatography (HIC) column. 8MTT was eluted from the HIC column by decreasing the NaCl concentration. 8MTT in the eluant of the HIC column was concentrated, and buffer exchanged into 20 mM HEPES, 200 mM NaCl, pH 7 using tangential flow filtration (50 kDa cutoff membrane) to 10 mg 8MTT/ml. The addition of 200 mM NaCl to the diafiltration buffer increased 8MTT solubility and minimized aggregation. As a final polish, the retentate in the formulation buffer was directly applied to an anion exchange membrane in a flow through mode, followed by sterile filtration of the product. This reduced the endotoxin levels from about 1000 E.U./mg to < 5 E.U./mg of 8MTT protein. Low endotoxin levels are a requirement for injectable products and the purified 8MTT was well under the allowable injection limit [24].

### Biological/biochemical properties of 8MTT

**Fig. 2.**
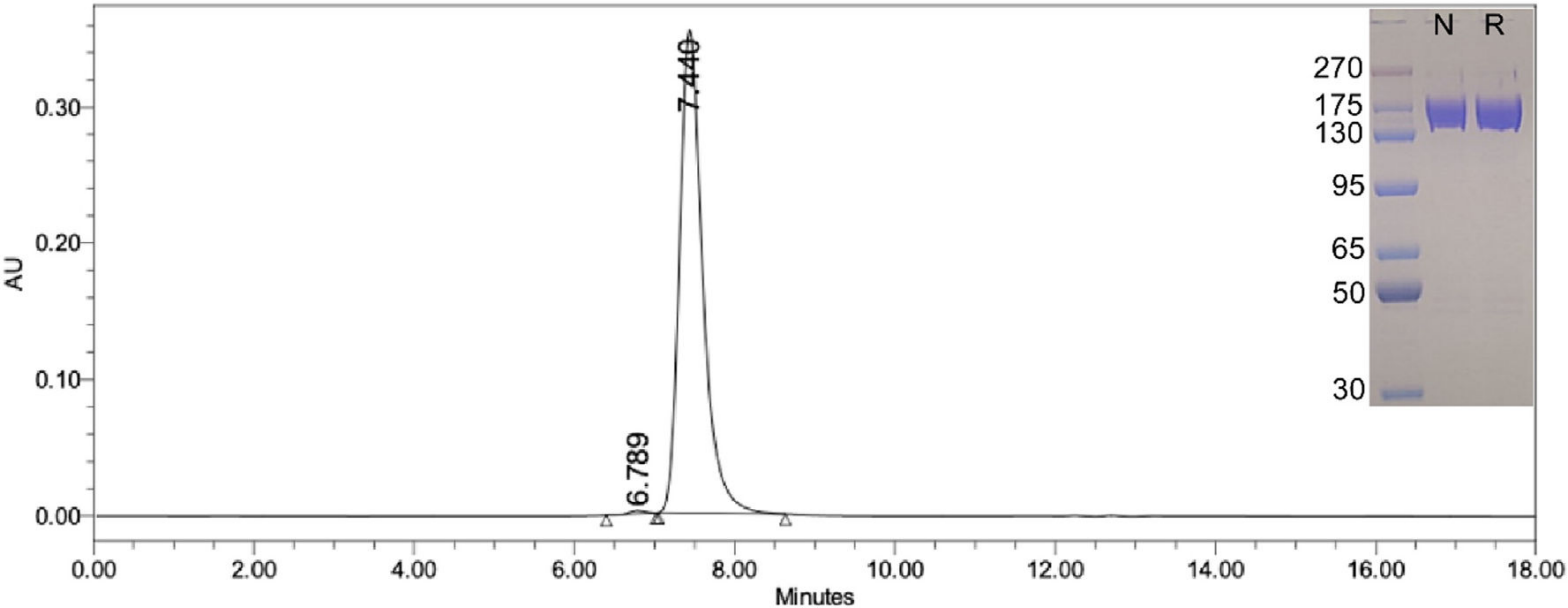
Purified 8MTT *SEC* HPLC was performed using a Waters Alliance system, using a Sepax *sEC*-150 column (3μ particle size, 7.8 × 300) equilibrated in PBS + 0.02 % sodium azide and run at 1 mL/min. Detection was at 280 nm (shown). (Insert) Purified 8MTT alone (oxidized) or in β-mer (reduced) was subjected to SDS-PAGE along with prestained-molecular weight marker proteins (270 kDa to 30 kDa), the gel was fixed, stained with Coomassie blue.

**Fig. 3.**
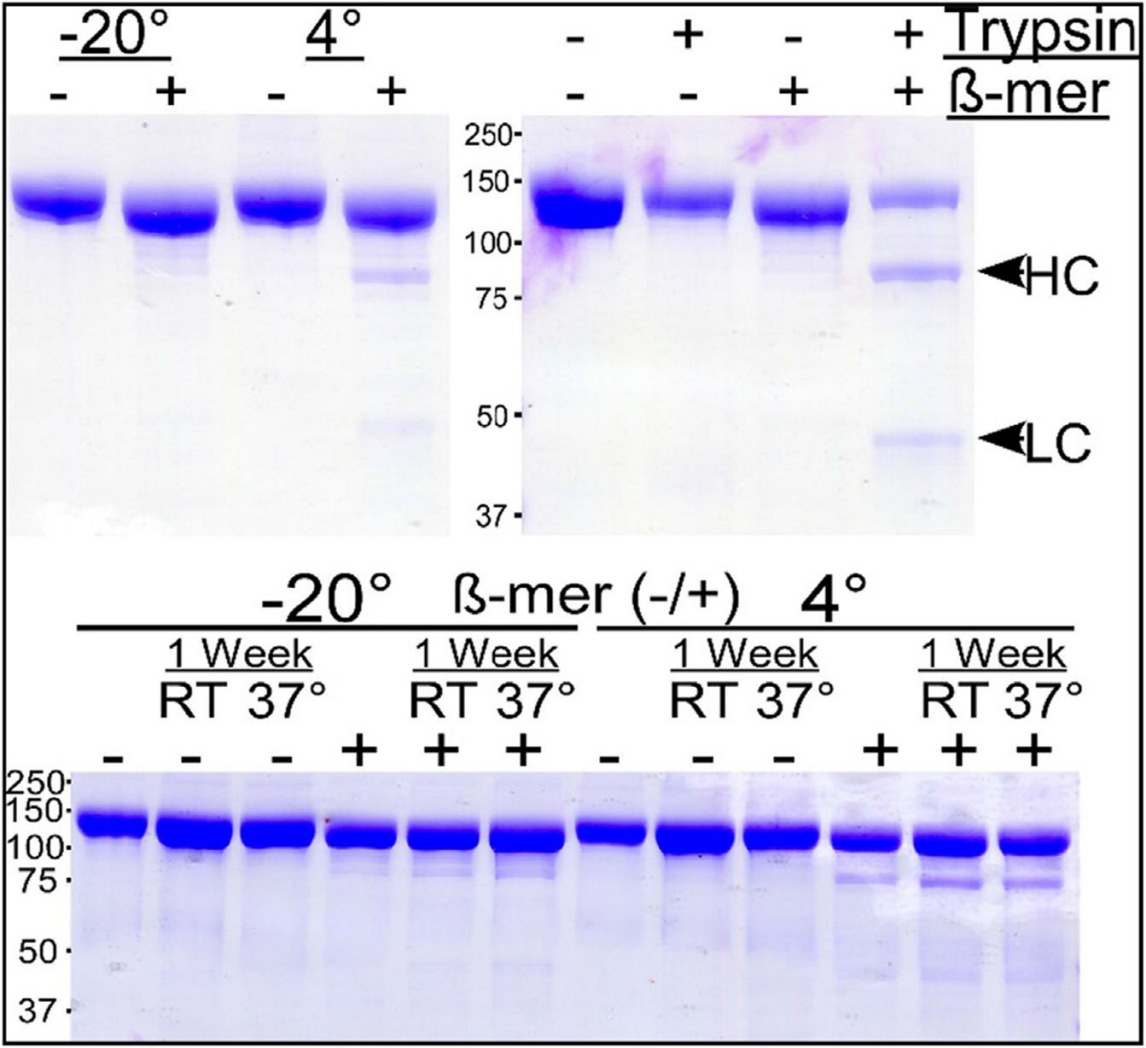
SDS-PAGE of Purified 8MTT stored for 1 year at −20 °C or 4 °C (UPPER panel) (Left gel) Purified 8MTT (10 μg) stored for 1 y at −20 °C or 4 °C. (Right Gel) Purified 8MTT (10 μg) stored for 1 y at −20 °C was incubated alone (−) or with trypsin (+), 1/1000 trypsin/8MTT, for 1 hr at 37 °C. (**LOWER panel**) Purified 8MTT (10 μg) stored for 1 y at −20 °C or 4 °C were incubated at room temp (RT) or 37 °C (37°) for 1 week. Samples were subjected to SDS-PAGE alone (−) or with β-mer (+). Gels were fixed and stained with Coomassie blue.

*SEC* HPLC of purified 8MTT appeared as a single peak with > 99 % purity and SDS PAGE exhibited a single band under both reducing and nonreducing conditions (Fig. 2). Tetanus toxin produced in *C. tetani* is a di-chain protein due to clostridial protease cleavage between the 50 kDa Light Chain (LC) and 100 kDa Heavy Chain (HC) and are held together by an interchain disulfide bond (C439-C467). By SDS-PAGE, purified 8MTT migrated as a ~ 150 kDa single band in either reducing or nonreducing conditions (Fig. 2), showing that the interchain disulfide bond was not cleaved during fermentation expression in Gor/Met^™^ *E. coli.* Native tetanus toxin is resistant to trypsin digestion, except for nicking between amino acids C439-C467 [25]. Purified 8MTT treated with trypsin migrated as a single-chain protein when analyzed by nonreducing SDS-PAGE, indicating retention of the disulfide bond between the Light Chain and Heavy Chain. When analyzed by reducing SDS-PAGE, two bands of the expected molecular weights for the LC and the HC were observed (Fig. 3 **Upper, Right Panel**). Thus, purified 8MTT retained the overall protein structure of native tetanus toxin and, like native tetanus toxin, was resistant to secondary cleavage by trypsin [18]. We interpret the presence of single chain 8MTT to mean *E. coli* does not possess a protease that can cleave 8MTT within the interchain region, rather than the cleavage site not being protease accessible, since trypsin preferentially cleaved 8MTT within the interchain region (Fig. 3). Future experiments will address the significance of 8MTT as a single chain protein relative the di-chain TTxd with respect to vaccine potency under standardized conditions.

To examine product stability, 8MTT was stored in buffer + 10 % glycerol for one year at −70 °C or −20 °C or 4 °C in buffer without glycerol. In addition, the ability to freeze 8MTT may be beneficial. We have not explored other cryoprotectants or if they are even necessary. *SEC* HPLC chromatograms of 8MTT stored at −70 °C or 4 °C overlaid on each other, with no additional peaks or aggregation evident. SDS-PAGE, under reducing and nonreducing conditions, showed that 8MTT stored in buffer + 10 % glycerol for one year at 4 °C or −20 °C remained a full-length, single-chain protein with only a trace of protease nicking (Fig. 3**, Upper, Left Panel**). Purified 8MTT stored for 1 year at 4 °C or −20 °C (−) and then incubated at room temp or 37 °C for one week showed little degradation (Fig. 3**, Bottom Panel**).

### Mass Spectrometry of 8MTT

**Fig. 4.**
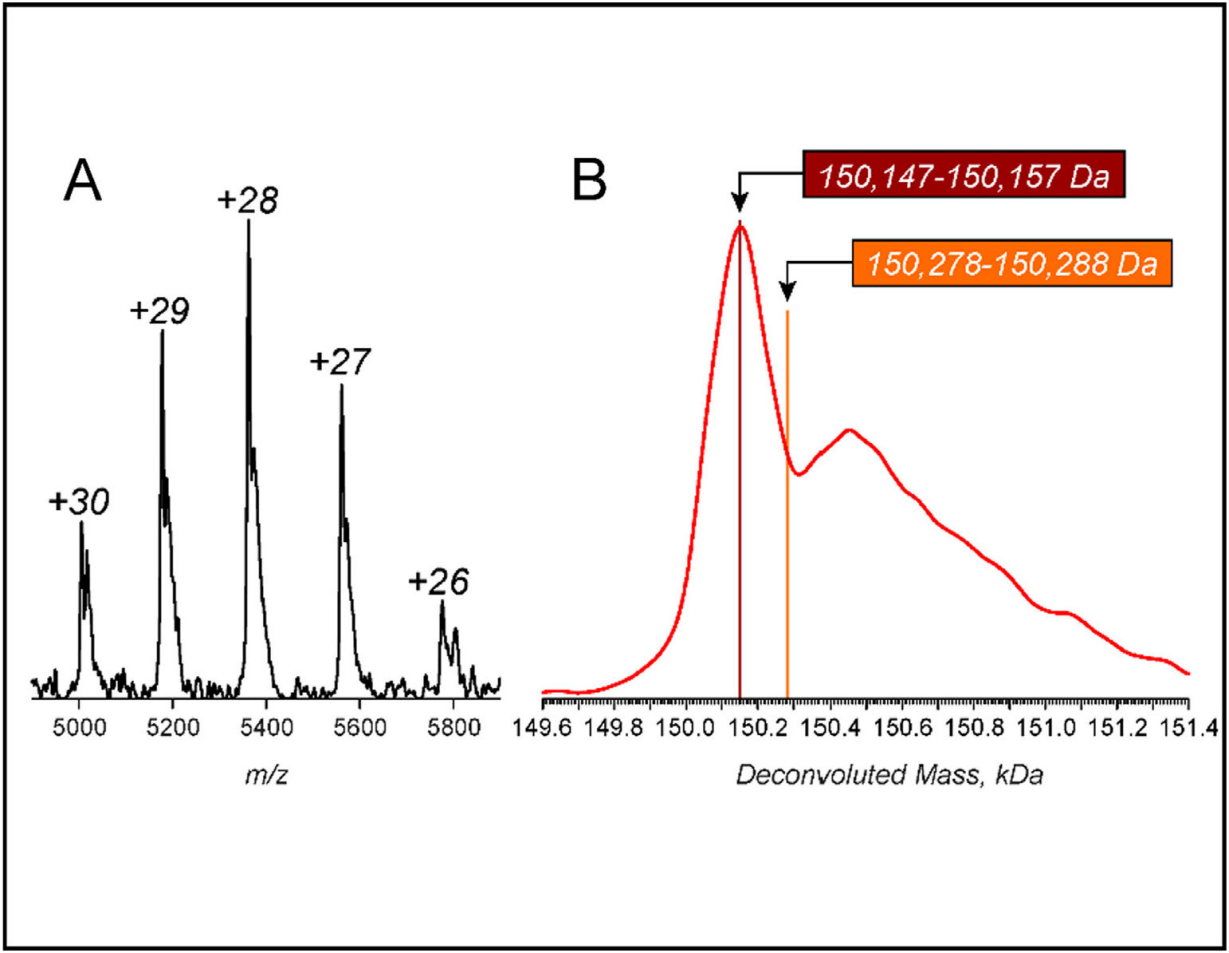
Mass Spectrometry of 8MTT A zoomed view of a native ESI mass spectrum of 8MTT showing the m/z region populated with the ionic signal (A), and the protein mass distribution deconvoluted from the MS data using the UniDec algorithm [25] (B). The protein ion charge states are labeled above each peak in panel A. The colored stripes in panel B indicate the mass ranges that can be populated by the putative protein species incorporating an *N*-terminal methionine residue (orange) and lacking it (maroon) depending on the oxidation status of their cysteine residues. The experimentally measured protein mass (at the apex of the main peak in the deconvoluted mass spectrum is 150,153 Da). The mass measured at the apex of the satellite peak (likely representing non-covalent adducts formed by the protein ions and polar solvent components – see the text for more detail) is 150,450 Da.

Purified 8MTT was analyzed by mass spectrometry (MS) to determine intact-mass and *N*-terminal proteolytic fragment composition. Intact-MS analysis (Fig. 4), performed under near-native conditions using electrospray ionization (ESI) to generate protein ions directly off the protein solution [26], showed a single protein species with a mass of 150,151 ± 6 Da. The mass of fully oxidized 8MTT, based on amino acid sequence (Swiss-Prot: P04958.2), is 150,147 Da and the mass with two disulfide bonds is 150,153 Da. No signal was detected for an 8MTT species that would include an *N*-terminal methionine (the calculated mass range 150,278 – 150,288 Da, depending on the oxidation status of the cysteine residues). Thus, the 8MTT mass spectrum is consistent with a protein that lacks an *N*-terminal methionine and has two disulfide bonds. Thus, the 8MTT mass spectrum is consistent with a protein that lacks an *N*-terminal methionine and has two disulfide bonds. The higher-mass peak (above 150,300 Da, apex at 150,450 Da) that is apparent in the deconvoluted spectrum likely represents adducts formed by association of the protein ins and polar components of the solvent in the gas phase, a phenomenon which is common in native MS [27]. We also noted that the ionic signal populated a high-*m/z* region of the mass spectrum, and that the observed charge state distribution was narrow (ranging from + 26 to + 30), indicating that 8MTT in solution maintained a compact conformation with no signs of unfolding and/or misfolding [28].

A second approach, a combination of MS measurements with protein fragmentation, further addressed the efficiency of the Gor/Met^™^ *E. coli* expression system to cleave the *N*-terminal methionine from 8MTT. 8MTT was initially analyzed by Orbitrap Fusion (Thermo-Fisher Scientific, Waltham, MA) MS with an integrated front-end reversed-phase (C18) NanoLC system, EASY-nLC^™^ 1000 (Thermo-Fisher Scientific, Waltham, MA) to obtain terminal peptide fragmentation. This identified a ladder of *N*-terminal peptides with the first residue of the sequence a Proline (Supplemental Fig. 1). No fragments were detected that corresponded to peptides with an *N*-terminal methionine. A more detailed structural characterization of a trypsin-generated peptide map of 8MTT was performed by LC/MS and LC/MS/MS analyses, which yielded a high-intensity signal for the *N*-terminal tryptic peptide PITINNFR (Supplemental Fig. 1). A signal was not detected for the corresponding *N*-terminal peptide that contained an *N*-terminal methionine residue, confirming the absence of a putative protein species that retained the initiation methionine. Thus, the Gor/Met^™^ *E. coli* strain efficiently removed the *N*-terminal methionine despite high 8MTT cytoplasmic expression.

Primary amines are often used for conjugation via NHS ester labeling and so the number of accessible ε lysine amines on a carrier protein were of interest. TNBS was used to estimate the number of primary amines on 8MTT [29]. Forty-seven primary amines per mole 8MTT were detected out of a possible 108. As a result of the formaldehyde toxoid process, we anticipated that tetanus toxoid would have many fewer available amines than 8MTT or the native toxin. However, evaluation of five monomeric preparations of tetanus toxoids, found 33–66 amines per mole TTxd, depending on the manufacturer and lot number [30]. Others have reported as few as 16 or as many as 88 amines per mole TTxd [31,32]. The wide range in primary amines among toxoids may reflect variations between manufacturers in the toxoid process, effects of aging, and/or the incorporation of media components into the vaccine. The presence of a specific number of modifiable amines, as found in recombinant proteins, such as 8MTT, would be an advantage for the reproducible synthesis of conjugate vaccines.

### Vaccine properties of 8MTT

Initial experiments determined the antigen content of 8MTT to be 4060 Lf/ml and the antigenic purity of 8MTT was 3500 Lf/mg protein nitrogen, above the recommended 1000 Lf/mg protein nitrogen specifications for human bulk purified tetanus toxoid vaccine components [33].

**Fig. 5.**
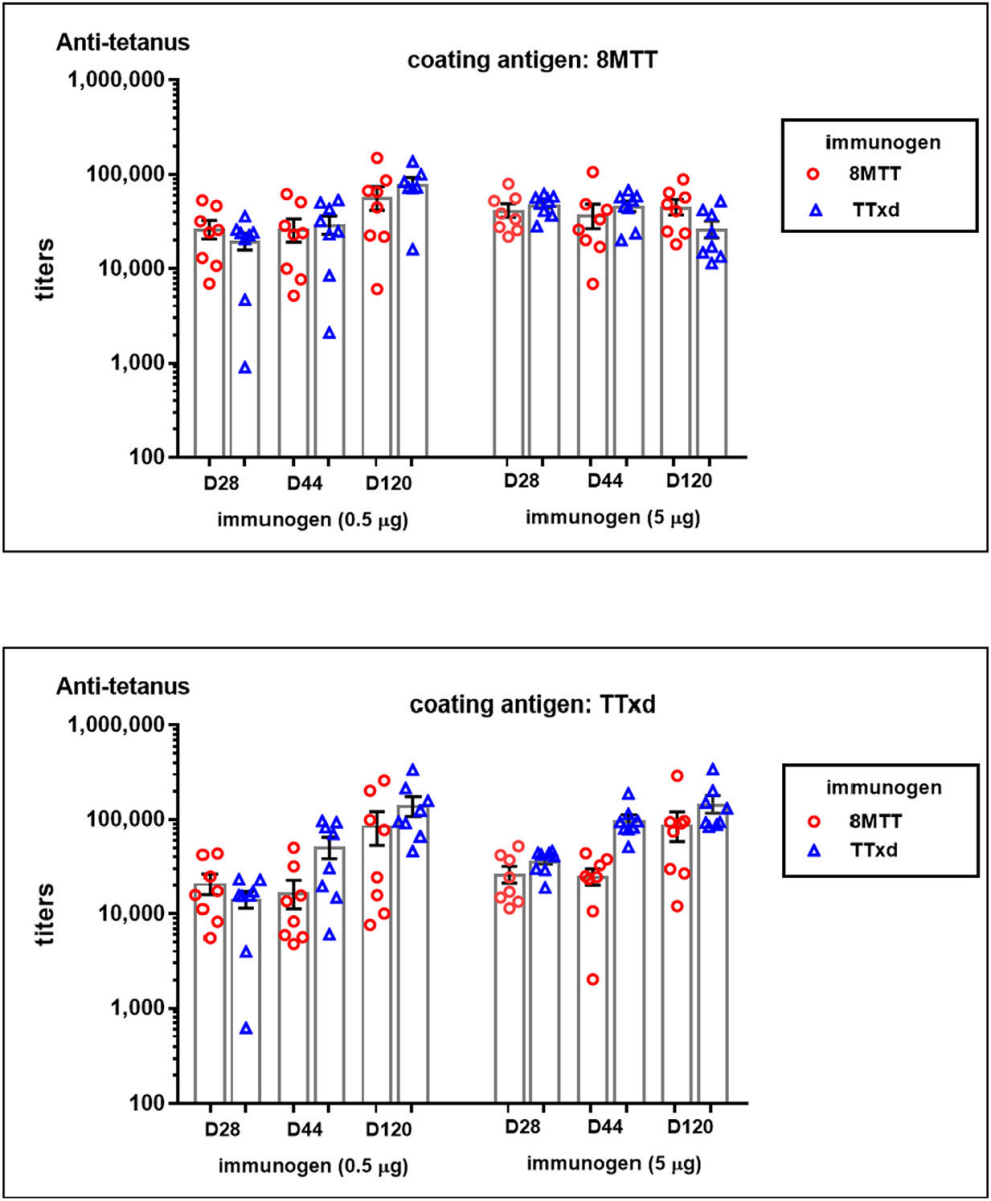
Anti-tetanus titers induced by 8MTT or TTxd vaccine CD-1 mice were immunized with 0.5 μg or 5.0 μg of 8MTT or TTxd on days 0, 14 and 110 and bled on days −1, 28, 44, and 120. anti-tetanus IgG were established with ELISA plates coated with either 8MTT (**Upper panel**) or TTxd (**Lower panel**) from the 28-, 44-, and 120-day bleeds.

We next evaluated purified 8MTT and purified TTxd as α-tetanus immunogens. Outbred CD-1 mice were immunized with a primary (day 0) and then boosted twice (day 14 and day 110) with 0.5 μg or 5 μg of 8MTT or TTxd, each absorbed to Alhydrogel^®^ aluminum adjuvant, and sera evaluated by ELISA using either 8MTT or TTxd as the coating antigen. 8MTT and TTxd each induced high and comparable levels of tetanus-specific antibodies, regardless of the ELISA coating antigen the unpaired *t*-test; two tailed P value was 0.008 (Fig. 5). Since earlier studies found α-8MTT or α-TTxd antibody titers correlated with the ability of 8MTT or TTxd immunized mice to neutralize a native tetanus toxin challenge in CD-1 mice [20] and that 8MTT possessed high antigenic purity, we extrapolate that 8MTT and TTxd elicited similar α-tetanus toxin neutralizing antibody titers.

### 8MTT as a conjugate vaccine carrier

**Fig. 6.**
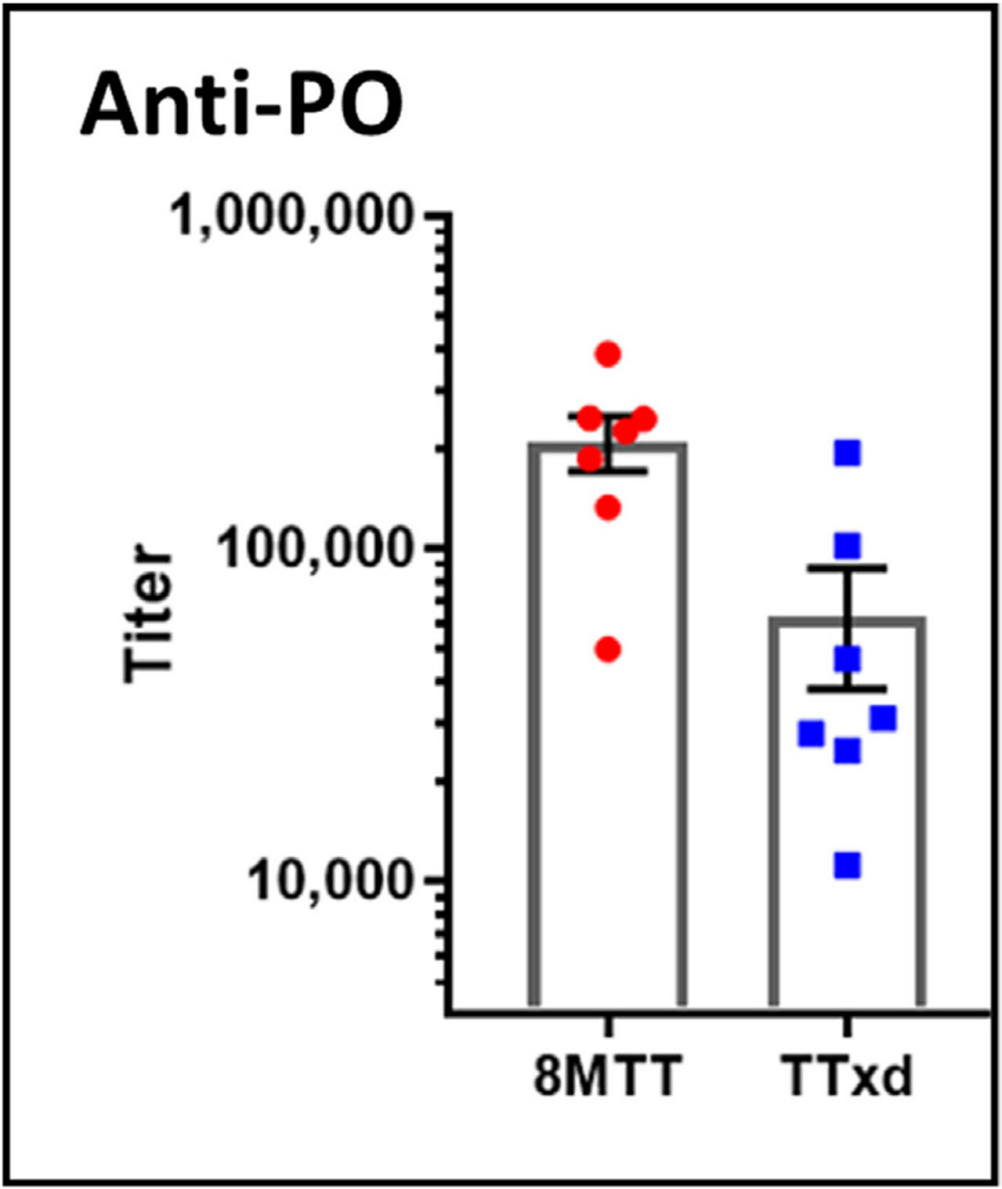
Anti-peptide P0 titers induced by conjugate P0-8MTT or conjugate P0-TTxd vaccines Balb/c mice were immunized with 2.5 μg of P0-8MTT or P0-TTxd on days 0 and 14 and bled on days −1 and 28. anti-P0 IgG titers were established with ELISA plates coated with P0-BSA from the 28-day bleed.

8MTT was next evaluated as a vaccine carrier for a peptide. The tick peptide, P0, under consideration as an immunogen for a cattle tick fever vaccine [34], was conjugated to 8MTT and monomeric TTxd. Balb/c mice were immunized subcutaneously with a primary (day 0) and then boosted on day 14 with 2.5 μg of conjugate (based on carrier protein). Sera from a day 28 bleed were assayed by ELISA for α-P0 peptide IgG titers, using a P0-BSA conjugate as the coating antigen. P0 peptide conjugated to either 8MTT or TTxd elicited statistically comparable α-P0 peptide IgG titers (Unpaired *t*-test), showing 8MTT to be at least as potent a conjugate carrier protein as TTxd for the P0 antigen (Fig. 6). Controls showed that immunization with the P0 peptide alone did not induce an anti-peptide IgG titer above the base line of Fig. 6.

**Fig. 7.**
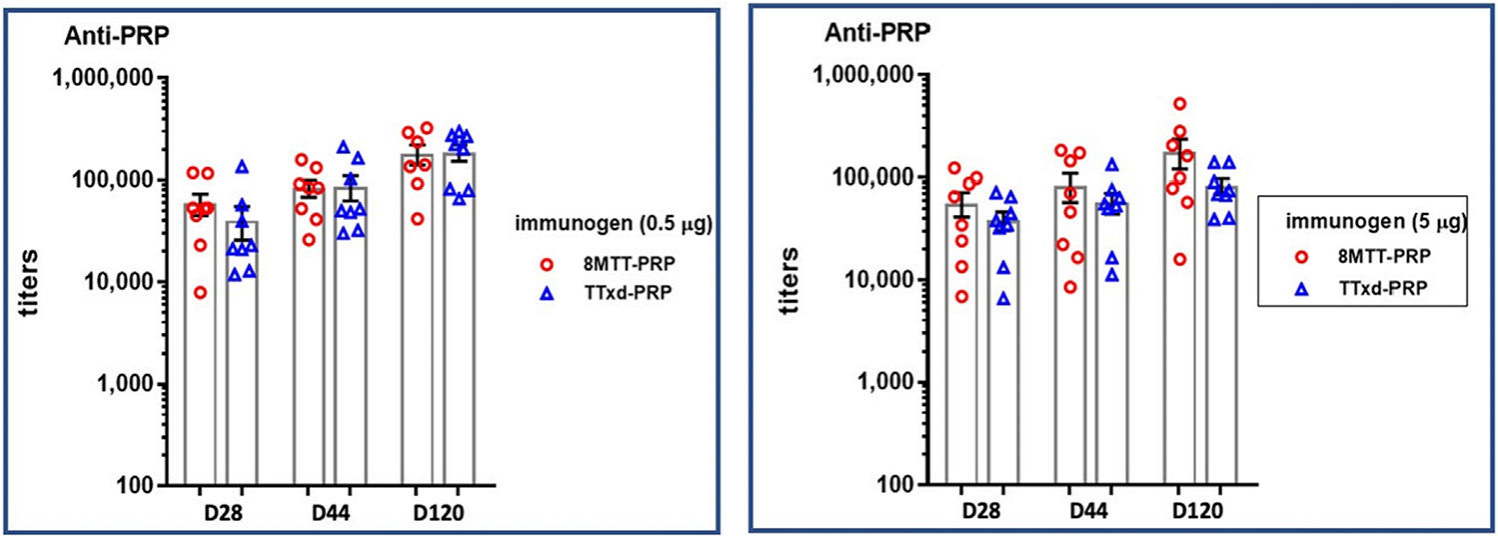
Anti-polysaccharide PRP titers induced by conjugate PRP-8MTT or conjugate PRP-TTxd vaccines. CD-1 mice were immunized with 0.5 μg or 5.0 μg of PRP-8MTT, PRP-TTxd or unconjugated PRP protein, representing 0.25 μg or 2.5 μg polysaccharide, respectively, on days 0, 14, and 110 and bled on days −1, 28, 44, and 120. anti-PRP IgG titers were established for the 0.5 μg (**Left panel**) or 5.0 μg (**Right panel**) of 8MTT-PRP, PRP-TTxd with ELISA plates coated with streptavidin followed by biotinylated PRP from the 28-, 44-, and 120-day bleeds.

8MTT and TTxd were next evaluated as conjugate vaccine carriers for polysaccharides, since polysaccharides, as *T*-cell independent antigens, are poorly immunogenic and do not show memory or class switching, limitations overcome by conjugation to a carrier protein [35]. We chose to compare the polysaccharide conjugate carrier potential for 8MTT using the capsular polysaccharide of *Haemophilus influenzae* b (PRP) since PRP-TTxd is a licensed conjugate vaccine for humans [36]. Using the same synthetic protocol, 8MTT and TTxd were each conjugated with ~ 0.5 μg of PRP polysaccharide per μg protein. Outbred CD-1 mice were immunized with a primary (day 0) and then boosted twice (day 14 and day 110) with 8MTT, TTxd, PRP-8MTT, PRP-TTxd or unconjugated PRP, normalized for 0.5 ug 5.0 ug of protein per inoculum, representing 0.25 μg or 2.5 μg PRP in the conjugate vaccine immunizations, respectively. Mice were bled on days −1, 28, 44, and 120. Sera were assayed for α-PRP IgG by ELISA. Both conjugate vaccines elicited similar high α-PRP antibody titers that were not statistically different, at either polysaccharide dose (Fig. 7). The different amounts of anti-TT IgG observed for 8MTT and TTxd at 44 days may reflect different epitopes between 8MTT and TTxd which could indicate TTxd being physically less uniform than 8MTT or TTxd and 8MTT have different epitopes that yield different decay rates of anti-TT IgG or a combination of the two outcomes. Mice immunized with 8MTT, TTxd, or unconjugated PRP did not elicit an α-PRP IgG antibody response above the baseline titer. Furthermore, as observed in Fig. 5 for immunization with 8MTT or TTxd alone, the α-carrier tetanus IgG titers were comparable for conjugated PRP-8MTTand PRP-TTxd, regardless of whether the ELISA coating antigen was 8MTT or TTxd (Supplemental Fig. 2). Thus, the antibody response elicited by PRP-8MTT to the tetanus and the PRP polysaccharide components were similar to the response induced by the PRP-TTxd. Also, immune response to 8MTT or TTxd when the immunogen was PRP-8MTT or PRP-TTxd (Supplemental Fig. 2) was similar to 8MTT or TTxd as immunogens alone (Fig. 5).

## 3. Discussion

Tetanus toxin (TT) is one of the most potent toxins known, and tetanus toxoid (TTxd) is a widely used clinical toxoid vaccine. We have developed a genetically detoxified, full-length tetanus toxin (8MTT) that is>50 millionfold less toxic than native tetanus toxin with retention of immunogenic potency relative to TTxd [37]. Here we describe the fermentation, production of 8MTT in Gor/Met^™^ *E. coli*, a novel strain engineered to have an oxidative cytoplasm and to efficiently cleave *N*-terminal methionine. 8MTT was expressed as a single chain protein without a purification tag and purified in two column steps to > 99 % purity with low endotoxin contamination, yielding 0.5 g of purified 8MTT / liter of fermentation culture, a notable accomplishment given that 8MTT is 150 kDa with ten cysteines, six of which are unpaired. Mass spectrometry confirmed the predicted molecular mass of 8MTT as a monomer and showed that the N terminus did not contain an *N*-terminal methionine and an immunological determination detected that 8MTT had antigenic purity of 3500 Lf/mg of 8MTT nitrogen by flocculation assay. We found 8MTT equally immunogenic to TTxd in mice as a tetanus vaccine and as a conjugate carrier protein.

A fair question is why there is a need for a new tetanus vaccine, given that the current tetanus toxoid is clinically effective, relatively inexpensive, and widely used. We propose that compared with the legacy tetanus toxoid vaccine, genetically detoxified tetanus toxin, 8MTT; (1) is easier, faster, and safer to manufacture, allowing 8MTT to be produced in standard bioprocessing facilities, (2) will be a vaccine of higher purity, which can be characterized/-standardized using modern techniques while minimizing animal use, and (3) is more suitable for use as a carrier protein for conjugate vaccines.

### Tetanus toxoid is a legacy vaccine made using methods that are poorly defined.

TTxd manufacturing (WHO/IVB/11.11) requires unique production considerations for laboratory safety and staff and environmental protection [38]. Fermentation of *C. tetani* for toxin production takes 7–10 days [39]. Although the WHO has long urged the use of a chemically defined media for *C. tetani* tetanus toxin production [40], establishing a chemically defined medium has been difficult to achieve while maintaining acceptable expression levels [41,42]. Yields of tetanus toxin in a recent publication were reported as ~ 100 Lf/ml, which we estimate ~ 100 Lf/ tetanus toxin per ml as ~ 0.2 g/L fermentation culture, using a value of 2 μg/Lf [39]. In contrast to the complexity of tetanus toxin production, 8MTT can be made in *E. coli*, a BSL1 organism, and produced in ~ 20 h using a standard bioprocessing fermentation facility. Our current efforts yielded about 0.5 g/L of purified 8MTT, using a chemically defined media supplemented with yeast extract. 8MTT yield should increase with further optimization but is already higher than toxoid production in *C. tetani*, which has seen many decades of process improvement.

First described by Ramon [43,44], formaldehyde is used to inactivate tetanus toxin in the spent culture medium of *C. tetani*. While purity has improved and current manufacturing protocols include tangential flow filtration and ammonium sulfate precipitation, the toxoided vaccine remains contaminated with *C. tetani* proteins and media components [10], and detoxification remains a lengthy 30- to 40- day incubation with formaldehyde at an elevated temperature. Chromatographic purification of tetanus toxin before detoxification has been applied by some manufacturers at large scale [32]. With eight mutations spanning the three-tetanus toxin functional domains, 8MTT does not require a detoxification step and can be purified using standard chromatography techniques. Genetic detoxification avoids the need to assay for residual formaldehyde as well as testing for reversion to toxicity.

### Historically, the safety and potency of legacy vaccines like TTxd require extensive animal testing for batch release.

The WHO requires the demonstration of the absence of toxicity of each TTxd batch, which often is performed in animals. However, the WHO has licensed a chemically inactivated TTxd based on an International Reference Reagent [33], where antigen content is normalized with a toxoid flocculation assay. Antigen content is determined as a visible flocculation of toxoid–antitoxin and expressed as Lf units [45], the number of units of antitoxin when mixed with a sample which produces an optimally flocculating mixture. Efforts are underway to develop *in vitro* assays for tetanus vaccine antigen content and to reduce animal use, testing tetanus vaccine potency [46]. These include attempts to quantify tetanus vaccine antigen content using antibody probes for critical epitopes [47,48] and peptide mapping [49]. To date, none of these alternatives have been implemented for batch release.

### Batch release based on consistent manufacture

The batch release for modern vaccines is based on understanding and controlling the manufacturing process, along with in-process and product testing using state-of-the-art analytical tools, allowing for the manufacture of a consistent product that can be compared to the material used for licensing [50]. Once the licensing lot has been approved, subsequent batches can be compared to the licensed lot without the need for additional extensive animal testing. The manufacture of a recombinant detoxified tetanus toxin is fully aligned with the “consistency approach” to modern vaccine production. 8MTT is produced as a recombinant protein in a standard BL21 *E. coli* strain using chemically defined media and can be characterized using a battery of biophysical, immunological, and biochemical techniques to define the product. We have begun to define the biophysical properties of 8MTT which would be necessary for a batch release. These include identification assays (SDS-PAGE, western blot, mass spectrometry, amino acid analysis, peptide mapping for sequence and disulfide bond formation), manufacture safety (endotoxin, host cell protein), aggregation (*SEC* HPLC), higher-order structure (CD, FTIR), thermal stability (ITC), along with toxicity assays.

### 8MTT as a new tetanus vaccine

Tetanus vaccine efficacy for 8MTT is, of course, a critical attribute. The WHO minimum antigenic purity of tetanus toxoid is 1000 Lf units per milligram of protein nitrogen [51,52]. Analysis of 8MTT using the international TTxd reference standard found the antigenic purity of 8MTT was a value of 3500 Lf /mg protein 8MTT nitrogen, suggesting that purified 8MTT potency was more than sufficient to be used as a tetanus vaccine. Our murine immunizations found that 8MTT induced comparable levels of α-tetanus IgG in mice as the TTxd. We did not find a difference in the antibody titers whether 8MTT or TTxd was used as the ELISA coating antigen, suggesting similar epitopes were immunogenic. An earlier 8MTT vaccine version, which contained a histidine tag, was found to induce tetanus toxin neutralizing antibodies [20]. At low dose immunizations, this earlier vaccine version was a potent vaccine like purified TTxd [20]. We anticipate 8MTT, as a more purified product, would be less reactogenic than a toxoided vaccine.

The receptor-binding domain of tetanus toxin (THc) has been evaluated as a recombinant tetanus vaccine [53] and as a carrier protein [54]. THc is about 1/3 the size of tetanus toxin, while 8MTT is a full-length mutated protein and encompasses all three tetanus toxin domains. THc induces protective antibodies to the toxin but antibodies against all the domains have been shown to provide better protection [55-57]. 8MTT is likely to provide superior protection against tetanus toxin than THc.

### 8MTT as a carrier protein for conjugate vaccines

Many antigens such as haptens, peptides, and carbohydrates are poorly immunogenic unless chemically linked to a carrier protein. Tetanus toxoid is used as a carrier protein in licensed conjugate vaccines for polysaccharides [51,58,59,60,61,62,63], peptides and haptens [64,65] and proteins [66] as well as many conjugate vaccines currently in development [67]. Due to aggregation and low purity, bulk tetanus toxoid is usually purified prior to conjugation, either by size exclusion chromatography or tangential flow filtration, to prepare a mostly monomeric conjugation grade fraction. We found that 8MTT was comparable to tetanus toxoid monomer as a carrier protein for a peptide, P0, and a polysaccharide, Hib PRP, inducing the same α-IgG titers against the peptide and polysaccharide, respectively, in mice. The response was long-lived and responsive to booster immunization. The purity of 8MTT is also higher than the minimum requirement for tetanus toxoid as a carrier protein (where the requirement is greater than tetanus vaccines at 1500 Lf/mg protein nitrogen). Furthermore, the α-8MTT and α-TTxd IgG responses of the respective conjugates (Supplemental Fig. 2) were statistically indistinguishable relative to the α-8MTT and α-TTxd IgG responses elicited by 8MTT and TTxd alone (Fig. 5), indicating that PRP did not squelch the immune response to either protein carrier, 8MTT and TTxd. Thus, 8MTT could complement TTxd as a licensed conjugate vaccine carrier protein [68,69].

The TTxd monomer fraction, although “purified,” is nevertheless a heterogeneous and variable product, increasing the challenge of manufacturing and characterizing conjugate vaccines [70]. In contrast to the variable amine-ratios found for tetanus toxoids, recombinant 8MTT is a homogeneous protein, facilitating the manufacture of a consistent conjugate product. Peptide-8MTT conjugates can be analyzed by standard mass spectrometry to determine a hapten:protein ratio, but this is not usually possible with the corresponding TTxd conjugate [71]. In addition to haptens and polysaccharides, some proteins are poorly immunogenic unless conjugated to a carrier protein and 8MTT can substitute for TTxd in this role [66]. As a recombinant protein, we anticipate the expression of 8MTT fusion proteins in the Gor/Met^™^ *E. coli* strain as an additional vaccine strategy and as an alternative to chemical conjugation, since 8MTT protein fusions mediate translocation of biologically active β-lactamase (27-kDa) across an endosome membrane [19,72].

## 4. Conclusion

There is a strong manufacturing and regulatory argument for bringing the tetanus vaccine into the 21st century with a genetically detoxified toxin. Our animal data suggests that 8MTT will be an efficacious stand-alone vaccine for tetanus and as a carrier protein. Still, we recognize the challenge to make the economic case for replacing the toxoid vaccine, as TTxd is a component of many licensed vaccines. Employment as a conjugate vaccine carrier will provide the opportunity to obtain the human data needed to expand the use of 8MTT as a vaccine for the toxin as well.

## 5. Methods

### Subclone the gene encoding 8MTT into Gor/Met^™^ *E. coli* expression system

An expression system was constructed for the production of 8MTT in the cytoplasm of Gor/Met^™^ *E. coli* [73]. Plasmid (p8MTT) was created by subcloning the 8MTT gene into pET24 plasmid with the T7 promoter replaced with a Tac promoter. p8MTT was then electroporated into BL21 Gor/Met^™^ competent cells. The gene encoding 8MTT on p8MTT was sequenced in multiple sequencing rounds to confirm the nucleotide sequence of *8mtt*. Alignment of 8MTT to the published 8MTT sequence [20] confirmed 100 % amino acid identity. Intracellular expression of the 8MTT was accomplished in the presence of Kanamycin upon induction with IPTG. We observed proper folding and disulfide bonding only with induction below 25 °C. The final BL21 cell line of Gor/Met^™^ strain (*fhuA2 lon ompT Δgor::map gal dcm hsdS*) containing the p8MTT expression vector was prepared and frozen as the research cell bank and utilized in the production of the master & working cell banks.

### Development of the Production Cell Line

One vial of parental cell line (BL21 Gor/Met^™^) was expanded and transformed with the p8MTT expression vector with one round of electroporation followed by plating for isolation on LB media with Kanamycin (50 μg/ mL). The next day, three individual colonies were picked, grown in 3 mL of non-inducing MDG Kanamycin medium [74] (25 mM Na_2_HPO_4_, 25 mM KH_2_PO_4_, 50 mM NH_4_Cl, 5 mM Na_2_SO_4_, 2 mM MgSO_4_, 0.5 % Glucose, 0.25 % Aspartate, 50 μg/ mL Kanamycin Sulfate, 0.2 X Trace Metals (4 μM CaCl_2_, 0.4 μM each of CoCl_2_, CuCl_2_, NiCl2, NaMoO_4_, H_3_BO_3_, and 2 μM ZnCl_2_), and 100 μM (NH_4_)_2_Fe(SO_4_)_2_). After 24 h of culture time at 37 °C resulting in an OD600 of 3, a 60 μl aliquot of the culture was removed and used to inoculate 3 mL of LB media with Kanamycin (50 μg/ mL) for expression analysis while the remaining culture was added 50/50 with 50 % glycerol and stored at −70 °C. Once 8MTT expression was confirmed by SDS-PAGE, as evidenced by banding at the correct molecular weight, the glycerol stocks of positive clones were selected for expansion into 100 mL MDG Kanamycin medium followed by incubation at 37 °C with 250 RPM shaking for 20 h. The culture was cryopreserved with 25 % (v/v) glycerol and aliquoted into 1 mL aliquots, thus creating the Research Cell Bank.

### Fermentation

We used fed-batch fermentation to grow 8MTT transformed bacteria. The seed culture was prepared by inoculating one glycerol vial of 8MTT transformed bacteria stock into 50 mL MDG media [74] and grown overnight in a 37 °C shaker Incubator at 250 rpm. The seed culture was used to inoculate 3L of fermenter media composed of 15 mM (NH_4_)_2_SO_4_, 80 mM K_2_HPO_4_, 25 mM Na_2_HPO_4_, 5 % glycerol, 40 μM CaCl_2_, 20 μM MnCl_2_, 4 μM CoCl_2_, 4 μM CuCl_2_, 4 μM NiCl_2_, 4 μM Na_2_MoO_4_, 4 μM H_3_BO_3_, 20 μM ZnCl_2_, 0.4 μM Na_2_SeO_3_, 2 mM MgSO_4_, 0.2 % Trace minerals, 0.1 mM (NH_4_)_2_Fe(SO_4_)_2_, 50 μg/ml kanamycin, and 0.01 % antifoam in a 5L vessel (New Brunswick Scientific Co. Inc). Fermentation was controlled by a Lab Owl Bioreactor Control System with the following parameters: 37 °C constant temperature, O_2_ saturation level (DO) set at 25 %, stirring speeds cascading from 300 to 800 rpm controlled by DO, pH 7.2 regulated by adding concentrated NH_4_OH, and constant air to oxygen (depending on DO) sparging flow rates at 2.5 L/ min. During first four h of growth, the DO slowly decreased and settled at the 25 % set point. The feed media (50 % glycerol, 5 % yeast extract, 20 mM MgSO_4_, 60 μM CaCl_2_, 30 μM MnCl_2_, 6 μM CoCl_2_, 15 mM (NH_4_)_2_SO_4_, 6 μM Na_2_SeO_3_, 6 μM H_3_BO_3i_ 30 μM ZnCl_2_, 0.1 mM (NH_4_)_2_Fe(SO_4_)_2_, and 50 μg/ ml kanamycin) was then added to the fermenter vessel at 1 mL/min. When the bacterial concentration reached an OD600 of 10, the temperature was lowered to 25 °C, followed by the addition of IPTG to a final concentration of 0.5 mM to induce 8MTT production. The feeding rate was then changed to a linear rate from 0.7 mL to 1.8 mL per min in 18 h. The fermentation was stopped when the feed media was completely consumed and the OD600 could reach 100. Cells were harvested by centrifugation at 10,000 × *g* for 10 min. Approximately 200 g wet pellets per L fermentation culture was obtained. The cells were frozen in flattened plastic bags and stored at −70 °C (frozen cell paste).

### Purification of fermentation produced 8MTT

Frozen cell paste was suspended in 20 mM Tris pH 7.0 (20 mL/ g paste) and allowed to thaw while stirring. Cells were broken in a Panda GEA homogenizer with a single pass at 16,000 psi. The whole cell lysate was clarified by centrifugation at 22,000 × *g* for 30 min followed by filtration with a 0.45 μm PES filter. The clarified soluble lysate was applied to an anion exchange column (Workbeads 40Q resin, Bio-Works, Uppsala, Sweden), washed with 75 mM NaCl in 20 mM Tris, pH 7.0 and 8MTT eluted with 150 mM NaCl in 20 mM Tris, pH 7.0. NaCl was then added to the Q eluant to a final concentration of 2 M and the eluent was applied to a Toyopearl^®^ Butyl-600 M column (Tosoh Biosciences), equilibrated with 2 M NaCl in 20 mM Tris pH 7.0. 8MTT was eluted with 1 M NaCl in 20 mM Tris, pH 7.0. The resulting eluant was concentrated and buffer exchanged into 20 mM HEPES, 200 mM NaCl, pH 7, using tangential flow filtration. The retentate was then passed through a Mustang Q membrane (Pall Life Sciences) and sterile filtered. For long term storage, purified 8MTT was made 10 % glycerol, aliquoted and frozen at −20 °C, or −70 °C.

### Characterization of purified 8MTT

SDS-PAGE was performed with 4–20 % bis-Tris gels (Genscript) followed by protein staining/destaining. *sEC*-HPLC was performed using a Waters Alliance system and a Sepax *sEC*-150 column (3μ particle size, 7.8 × 300), equilibrated with PBS + 0.02 % sodium azide and run at 1 mL/min. Detection was at 280 nm. Primary amine concentrations were determined using trinitrobenzene sulfonic acid (TNBS) [29]. Lf assay was performed at the National Institute for Biological Standards and Control (United Kingdom), using the 3rd International Standard tetanus toxoid for use in flocculation test as the reference standard and 66/021 Equine tetanus antitoxin as the flocculation antibody.

### Mass Spectrometry of 8MTT

MS characterization of the purified protein was carried out both at the intact-mass level and the peptide map level. The former was carried out under near-native conditions, following the protein transfer to 150 mM ammonium acetate, pH 7.1 via extensive buffer exchange using an Amicon Ultra-4 30 kDa cutoff centrifugal filter (Millipore-Sigma, St. Louis, MO). A SolariX 7 (Bruker Daltonics, Billerica, MA) Fourier transform ion cyclotron resonance MS equipped with a conventional ESI source and a 7 T superconducting magnet was used for the intact-mass measurements. The peptide mapping was carried out using an Orbitrap Fusion (Thermo-Fisher Scientific, Waltham, MA) MS with an integrated front-end reversed-phase (C18) NanoLC system, EASY-nLC^™^ 1000 (Thermo-Fisher Scientific, Waltham, MA). The protein was reduced with dithiothreitol, alkylated with iodoacetamide and digested with a proteomics-grade trypsin (Sigma-Aldrich, St. Louis, MO) for 24 h at room temperature using a 1:100 trypsin/substrate ratio. The initial identification of tryptic fragments was carried out by searching the entire complement of peptide ions for the monoisotopic masses falling within 15 ppm of the values that were calculated based on the sequence. The peptide ion identities were then confirmed based on the fragmentation patterns generated by collision-induced dissociation (MS/MS measurements). The details are provided in Supplemental Fig. 1.

### Production of Conjugate vaccines

Tetanus toxoid (obtained from Panacea, New Dehli, India) was fractionated using Super-dex200 size exclusion chromatography to prepare a monomeric fraction. ***Peptide P0*** (AAGGGAAAAKPEESKKEEAK) is derived from an immunogenic region of ribosomal protein P0 of Rhipicephalus spp. that is being evaluated as a broad-spectrum α-tick vaccine [75]. A chemical conjugate of the tick P0 peptide was found to be efficacious against *Amblyomma mixtum*. P0 was synthesized with an *N*-terminal cysteine and prepared to > 98 % purity by Peptide 2.0 (Chantilly, VA). 8MTT and TTxd were reacted with a 50-fold molar excess of the maleimide reagent GMBS in 0.1 M HEPES, pH 7.2. At 1 hr, the solution was desalted with an Amicon Ultra 30 kDa cutoff centrifugal device using PBS, pH 6.8. Cysteine-pep tide:maleimide-protein were combined at a 40:1 M ratio at pH 7.2. Following an overnight incubation at 4 °C, unconjugated peptide was removed by extensive dialysis against PBS using a membrane with a 7 kDa cutoff. The product was concentrated using an Amicon device and sterile filtered. ***PRP polysaccharide*** (obtained from the Serum Institute of India, Pune, India) was aminated using CDAP cyanylation reagent, essentially as described [76]. In brief, to 6 mL of aqueous PRP (3 mg/ml) on ice, 300 μl of 2.5 M dimethylaminopyridine, pH 7.5 was added, titrating with 0.1 M HCl to adjust the pH to 8. To start the activation, 250 μl of 100 mg/ ml CDAP in acetonitrile was added to the stirred solution. The pH was maintained between 7 and 8 by the addition of 10 μl aliquots of 0.1 M NaOH. After 90 min, six ml of 1 M Hexane diamine in 0.1 M HEPES, pH 8 was added. After 1 h reaction time, reagent was removed by extensive dialysis, first against 1 M NaCl and then water. The amino-PRP was then concentrated to 3 mL, using an Amicon Ultra 15 device (30 kDa cutoff). The amine concentration was determined using the TNBS assay with glycine as the amine standard [29] and a resorcinol/sulfuric acid assay with ribose as the standard [29], with a conversion factor of 331g PRP/- mole ribose. 8MTT and monomeric TTxd were thiolated with SPDP (Covachem, LLC, Loves Park, IL) using 15x molar excess. Following deprotection and desalting, a ratio of 16 and 11 thiols/mole 8MTT and TTxd, respectively were determined. Amino PRP was converted to maleimide PRP using excess GMBS, pH 7.2. After a 1 h reaction, excess reagent was removed using an Amicon Ultra 15 device (30 kDa). Thiol-protein and maleimide-PRP were combined at a 1:1 wt ratio and allowed to react for 2 hr at room temperature followed by 18 hr at 4 °C. The reaction was quenched by the addition of 0.1 mM mercaptoethanol. Each conjugate was purified using a Superdex 200 size exclusion column, equilibrated with PBS. The conjugate fraction was pooled and sterile filtered. Protein was quantitated using the microBCA assay (Thermofisher) with 8MTT as the standard, while carbohydrate was determined using a resorcinol/sulfuric acid assay [77] with ribose as the standard. A conversion of 331 g/mole PRP per mole ribose was used. Note, 0.5 μg or 5 μg of PRP-8MTT or PRP-TTxd immunizations contained 0.25 μg and 2.5 μg of PRP, respectively.

### Immunizations

Antigens were prepared by combining with Alhydrogel^®^ such that each dose would contain 1 mg aluminum adjuvant in 100 μl of PBS. Two mouse strains are used; CD-1 mice for the vaccine and polysaccharide immunizations and Balb/c mice which were used for PO immunizations, based upon prior use of this mouse strain with the PO antigen. ***PO-8MTT and P0-TTxd Immunizations*** P0 peptide-8MTT and P0 peptide-TTxd conjugates were evaluated in Balb/c mice. For the P0 conjugate study, groups of 8 Balb/c female mice were immunized subcutaneously with 2.5 μg of conjugate (based on protein) on days 0 and 14. Sera from *retro*-orbital bleeds were taken on days −1 and 28. ELISA plates were coated with P0-BSA, and sera analyzed for anti-P0 IgG (described below). ***8MTT, TTxd, PRP-8MTT and PRP-TTxd Immunizations*** 8MTT, TTxd, PRP-8MTT, PRP-TTxd, or PRP alone were evaluated in ICR mice. For the 8MTT and TTxd and PRP-8MTT and PRP-TTxd studies, groups of 8 ICR female mice were immunized subcutaneously with 0.5 μg or 5 μg of 8MTT, TTxd, PRP-8MTT or PRP-TTxd on days 0, 14 and 110. Sera from *retro*-orbital bleeds taken on days −1, 28, 44, and 120. Note, 0.5 μg or 5 μg of PRP-8MTT or PRP-TTxd immunizations contained 0.25 μg and 2.5 μg of PRP, respectively. ELISA plates were coated with either 8MTT or TTxd and sera analyzed for anti-tetanus IgG, while ELISA plates were plated with streptavidin/biotinylated PRP, and sera assayed for anti-PRP IgG (described below).

Immunizations was carried out by Noble Life Sciences, German-town, MD in compliance with the current version of the following 1) Animal Welfare Act Regulations (9 CFR); 2) U.S. Public Health Service Office of Laboratory Animal Welfare (OLAW) Policy on Humane Care and Use of Laboratory Animals; 3) Guide for the Care and Use of Laboratory Animals (Institute of Laboratory Animal Resources, Commission on Life Sciences, National Research Council, 1996); and 4) AALAC accreditation.

### Immunoassays

For each assay, titers were determined using the formula: EXP(((LN(B)-LN(A))/(D-C))*(E-C) + LN(A)), where; E = Titer Point (a chosen absorbance value, usually ≥ 1.000; A = dilution giving an absorbance value above the titer point; B = dilution giving an absorbance value below the titer point;C = absorbance value at dilution A and; D = absorbance value at dilution B. ***Detection of antibodies reactive with 8MTT or TTxd***: Greiner High Bind immunoassay plates were coated with 100 μl per well of 8MTT or TTxd at 1 mg/ ml in PBS. After overnight incubation at room temperature, plates were washed once with 300 μl of PBS and 180 μl/well of Casein Blocking Solution (Surmodics) was added to each well and the plates were incubated for 60 min at room temperature. Plates were washed 3X with PBS containing 0.05 % Tween 20 (PBS-T) and serum from each mouse (diluted in PBS-T from 1:100 to 1:1562500) was added to duplicate wells. The plates were incubated for 60 min at room temperature and washed as noted above. One hundred μl of goat anti-mouse IgG Fc – HRP (Southern Biotechnologies), diluted 1:20000 in PBS-T, was added to each well. The plates were again incubated for 60 min at room temperature and washed with PBS-T. One hundred μL of TMB substrate (Moss Substrates) was added per well and plates incubated in the dark for 15 min. Reactions were stopped by addition of 100 μl of 0.5 N HCl per well and the absorbance at 450 nm were determined using a Molecular Devices Vmax plate reader. The results of 8MTT or TTxd immunizations are shown in Fig. 5. ***Detection of antibodies reactive with P0:*** anti-peptide P0 antibodies were detected using the above protocol for the 8MTT/TTxd, but with the ELISA plates coated with P0-BSA. Following an overnight incubation, the assay was continued as described above for 8MTT/TTxd assay. The results of P0-8MTT or P0-TTxd immunizations are shown in Fig. 6 (anti-P0 IgG). ***Detection of antibodies reactive with PRP:*** Greiner High Bind immunoassay plates were coated with 100 μl per well of streptavidin solution (1 mg/ml). After overnight incubation at room temperature the plates were washed 3X with PBS-T and 100 μl of biotinylated PRP (2 μg/ml; Fina Biosolutions) was added to all wells. Following a 60 min incubation, the assay was continued as described above for 8MTT/TTxd assay. The results of PRP-8MTT or PRP-TTxd immunizations are shown in Fig. 7 (anti-PRP IgG) and Supplemental Fig. 2 (anti-tetanus IgG).

### Statistical Analysis

Data were analyzed for statistical significance, using a *t* test for grouped sera and an ordinary-one-way analysis of variance (ANOVA) assuming normal distribution vaccine experiments, using GraphPad Prism 7.

## Supplementary Material

Supplementary Material
