# Supplementary material for: Genetically detoxified tetanus toxin as a vaccine and conjugate carrier protein
Source: Vaccine. Author manuscript; Available in PMC 2023 Jul 12. (PMC10336728; doi:10.1016/j.vaccine.2022.07.011)
Supplement: Supplementary Material [file NIHMS1909401-supplement-Supplementary_Material.docx]

**Supplemental Figure 1**


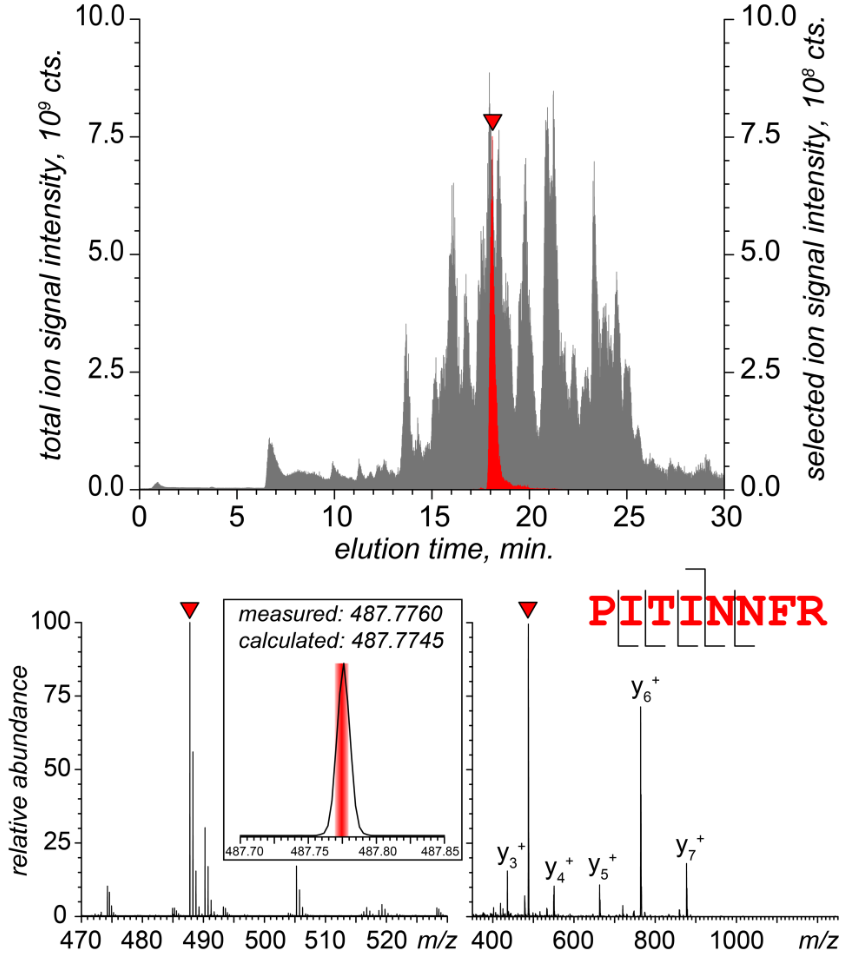


**Supplemental Figure 1**. Identification of the N-terminal sequence of 8MTT using peptide mapping and high-resolution LC/MS/MS detection. (**Upper panel**) The total ion chromatogram (gray) of the tryptic digest of 8MTT and the reconstructed ion chromatogram (red) of all ionic species whose masses fall within 15 ppm of 487.7745 Da (the calculated mass of a doubly protonated form of the predicted N-terminal tryptic fragment of 8MTT, PITINNFR). No ionic species representing a putative N-terminal peptide MPITINNFR were detected at this level of resolution/mass accuracy. (**Lower, Left panel**) A mass spectrum averaged across the 18.0-18.5 min elution window; the inset shows a zoomed view of a monoisotopic peak of a doubly charged tryptic fragment representing the N-terminus of 8MTT (this peak is labeled with a red triangle in the full-range mass spectrum). (**Lower, Right panel)** Fragmentation of the ion at *m/z* 487.4460 following its collisional activation generates a series of abundant singly charged *y*-ions confirming the sequence of this peptide as PITINNFR.

**Supplemental Figure 2**

**
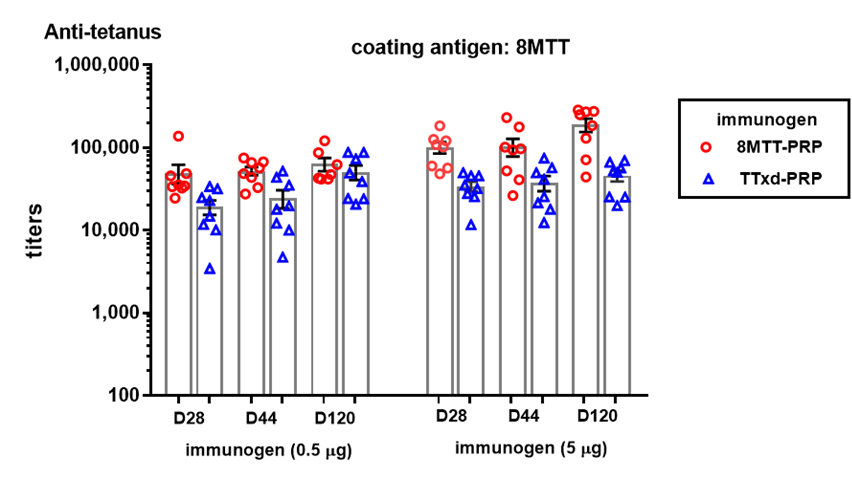
**


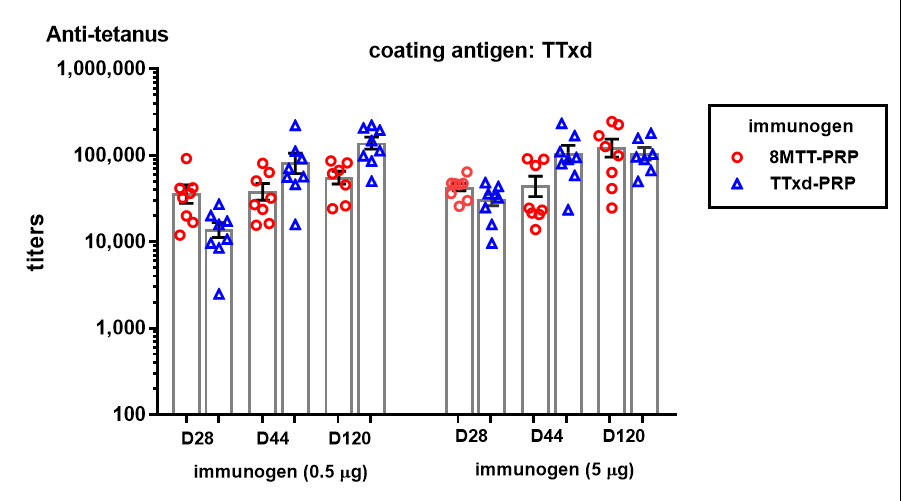


**Supplemental Figure 2. Anti-tetanus titers induced by conjugate PRP-8MTT or conjugate PRP-TTxd vaccines.** ICR mice were immunized with 0.5 μg or 5.0 μg of 8MTT-PRP, PRP-TTxdor unconjugated PRP protein, representing 0.25 µg or 2.5 µg polysaccharide, respectively, on days 0, 14, and 110 and bled on days -1, 28, 44, and 120. Anti-tetanus IgG titers were established for the 0.5 μg or 5.0 μg of 8MTT-PRP, PRP-TTxdwith ELISA plates coated with 8MTT (**Upper panel**) or TTxd (**Lower panel**) from the 28, 44, and 120 day bleeds.
